# Acid-sensing ion channel 3 is required for agmatine-induced histamine-independent itch in mice

**DOI:** 10.3389/fnmol.2023.1086285

**Published:** 2023-03-01

**Authors:** Guo-Kun Zhou, Wen-Jing Xu, Yi Lu, Yan Zhou, Chen-Zhang Feng, Jiang-Tao Zhang, Shi-Yu Sun, Ruo-Meng Wang, Tong Liu, Bin Wu

**Affiliations:** ^1^Institute of Pain Medicine and Special Environmental Medicine, Nantong University, Nantong, Jiangsu, China; ^2^Jiangsu Key Laboratory of Neuropsychiatric Diseases, Institute of Neuroscience, Soochow University, Suzhou, China; ^3^State Key Laboratory of Neuroscience, Center for Excellence in Brain Science and Intelligence Technology, Chinese Academy of Sciences, Institute of Neuroscience, Shanghai, China; ^4^College of Life Sciences, Yanan University, Yanan, China; ^5^Suzhou Key Laboratory of Intelligent Medicine and Equipment, Suzhou, China

**Keywords:** itch, agmatine, ASIC3, atopic dermatitis, pain

## Abstract

**Introduction:**

Itch is a common symptom of many skin and systemic diseases. Identifying novel endogenous itch mediators and the downstream signaling pathways involved will contribute to the development of new strategies for the treatment of chronic itch. In the present study, we adopted behavioral testing, patch clamp recording and metabonomics analysis to investigate the role of agmatine in itch and the underlying mechanism.

**Methods:**

Behavioral analysis was used to evaluate the establishing of acute and chronic itch mice model, and to test the effects of different drugs or agents on mice itch behavior. Western blotting analysis was used to test the effect of agmatine on phosphorylation of ERK (p-ERK) expression in the spinal cord. Patch clamp recording was used to determine the effect agmatine on the excitability of DRG neurons and the role of ASIC3. Finally, the metabonomics analysis was performed to detect the concentration of agmatine in the affected skin under atopic dermatitis or psoriasis conditions.

**Results:**

We fused a mouse model and found that an intradermal injection of agmatine (an endogenous polyamine) into the nape of the neck or cheek induced histamine-independent scratching behavior in a dose-dependent manner. In addition, the ablation of nociceptive C-fibers by resiniferatoxin (RTX) abolished agmatine-induced scratching behavior. However, agmatine-induced itch was not affected by the pharmacological inhibition of either transient receptor potential vanilloid 1 (TRPV1) or transient receptor potential ankyrin 1 (TRPA1); similar results were obtained from TRPV1^−/−^ or TRPA1^−/−^ mice. Furthermore, agmatine-induced itch was significantly suppressed by the administration of acid-sensing ion channel 3 (ASIC3) inhibitors, APETx2 or amiloride. Agmatine also induced the upregulation of p-ERK in the spinal cord; this effect was inhibited by amiloride. Current clamp recording showed that the acute perfusion of agmatine reduced the rheobase and increased the number of evoked action potentials in acute dissociated dorsal root ganglion (DRG) neurons while amiloride reversed agmatine-induced neuronal hyperexcitability. Finally, we identified significantly higher levels of agmatine in the affected skin of a mouse model of atopic dermatitis (AD) when compared to controls, and the scratching behavior of AD mice was significantly attenuated by blocking ASIC3.

**Discussion:**

Collectively, these results provide evidence that agmatine is a novel mediator of itch and induces itch via the activation of ASIC3. Targeting neuronal ASIC3 signaling may represent a novel strategy for the treatment of itch.

## Introduction

Itch is an unpleasant sensation that evokes the desire or reflex to scratch (Ikoma et al., 2006). Acute itch is normally a protective response that may help to remove irritants from the skin (Lay and Dong, 2020). In contrast, chronic itch lasts for more than 6 weeks and seriously reduces a patient’s quality-of-life and lacks effective treatment options. Chronic itch is commonly accompanied by various skin disorders (e.g., psoriasis and atopic dermatitis) and systemic diseases, such as liver diseases, kidney diseases, metabolic disorders, and cancers (Yosipovitch and Bernhard, 2013). Itch is closely related to pain. Both itch and pain stimuli may target a similar subpopulation of primary sensory neurons that is intrinsically multimodal; the same or different ligands may bind to different receptors to induce distinct behavioral patterns (Hu et al., 2021).

Based on the pathological mechanisms involved, itch is usually categorized as either histaminergic and histamine-independent itch (Dong and Dong, 2018). Various receptors or ion channels, including histamine receptors, serotonin receptors (Tian et al., 2016), Mas-related G protein-coupled receptor (Mrgpr; Kothari and Dong, 2022), opioid receptors (Snyder et al., 2018), cytokine receptors (Jing et al., 2018, 2019), toll-like receptors (Liu et al., 2010, 2016) and transient receptor potential (TRP) ion channels (Sun and Dong, 2016; Zhang et al., 2020; Wilzopolski et al., 2021) are expressed by primary sensory neurons in the dorsal root ganglia (DRGs) or trigeminal ganglions (TGs). These structures detect different exogenous or endogenous pruritogens and play an indispensable role in the occurrence and conduction of itch signaling. Histamine is released by mast cells and keratinocytes and acts on histamine H1 and H4 receptors to elicit scratching mainly by activating TRPV1 (Shim et al., 2007). Thus, antihistamines are frequently used for the treatment of itch in the clinic. The Mrgprs family, as the well-characterized histamine-independent itch receptors, can be activated by many endogenous and exogenous pruritogens, such as chloroquine, substance P and bovine adrenal medulla (BAM) 8–22 (Klein et al., 2021; Aliotta et al., 2022). Notably, the majority of chronic itches are insensitive to antihistamine treatment, thus suggesting the existence of histamine-independent mechanisms and distinct pruritogens that have yet to be discovered. Therefore, the underlying mechanism responsible for chronic itch has yet to be fully elucidated.

Acid-sensing ion channels (ASICs) belong to the epithelial sodium channel/degenerin (ENaC/DEG) family and are activated by extracellular protons. At least six ASIC subunits (ASIC1a, 1b, 2a, 2b, 3, and 4) form either homotrimeric or heterotrimeric channel complexes (Wemmie et al., 2006). The subunits of ASICs are distributed in a tissue-specific manner and exert different physiological functions (Deval and Lingueglia, 2015). ASIC3 is abundantly expressed in peripheral sensory neurons in DRGs (specifically on nociceptors) and has been where implicated in pain, chemosensation and mechanosensation (Li and Xu, 2011). ASIC3 generates a persisting current in response to moderate acidification (Salinas et al., 2009). In addition to extracellular protons, ASIC3 can also be activated by non-proton ligands, including 2-guainidinie-4-methylquinazoline (GMQ) and chloroquine. GMQ causes the persistent activation of ASIC3 at neutral pH (Yu et al., 2010). Due to the newly identified non-proton ligand-sensing domain, chloroquine has been found to selectively enhance the sustained phase of the ASIC3 currents which were involved in itch sensation in mice (Lei et al., 2016). Serotonin (5-HT), a classic inflammatory mediator, is also known to act on ASIC3 and facilitates acid-induced peripheral pain sensitivity (Wang et al., 2013). Notably, ASIC3-deficient animals were found to exhibit reduced scratching behavior induced by an MrgprC11 agonist, thus suggesting that ASIC3 may play an important role in histamine-independent itch (Peng et al., 2015). Together, the ASICs expressed on primary sensory neurons are critical for the signal transduction of pain and itch.

Agmatine is a biogenic cationic polyamine that is synthesized by the decarboxylation of L-arginine by arginine decarboxylase (ADC). Agmatine mainly exists in the form of a cation at physiological pH in bacteria, plants, invertebrates and mammals (Reis and Regunathan, 2000; Halaris and Plietz, 2007). Agmatine has been shown to exert modulatory actions on multiple molecular targets, including neurotransmitter receptors, membrane transporters, key enzymes and ion channels (Piletz et al., 2013). In the nervous system, agmatine has also been considered as a putative neurotransmitter that exhibits multi-receptor affinity and performs diverse physiological roles in the brain and in the periphery. For instance, agmatine binds to α2-adrenergic receptor and imidazoline receptor binding sites and blocks the NMDA receptor and other cation ligand-gated channels (Reis and Regunathan, 2000; Zhu et al., 2003; Wang et al., 2006). Recent research has shown that agmatine acts as an non-proton ligand for the ASIC3 channel and enhances ASIC3 currents (Li and Xu, 2011). However, the role of agmatine in itch remains unclear.

In the present study, we tested the hypothesis that agmatine may cause itch in mice by activating ASIC3 in DRG neurons. We found that an intradermal injection of agmatine induced histamine-independent itch in a dose-dependent manner in a mouse model. Furthermore, we found that ASIC3 was indispensable in agmatine-induced itch and the hyper-excitability of DRG neurons. Thus, our results suggest that ASIC3 signaling is critical for agmatine-induced itch and that targeting ASIC3 may serve as an important strategy for the treatment of chronic itch.

## Materials and methods

### Animals

Male ICR mice and C57BL/6 mice (6–8 weeks old) were obtained from the Shanghai SLAC Laboratory Animal Co., Ltd (Shanghai, China). Male *TRPA1*^−/−^and *TRPV1*^−/−^ mice were purchased from the Jackson Laboratory (Bar Harbor, ME, United States). All animals were housed under a 12-h light/dark cycle with food and water available *ad libitum*. The room was maintained at 22 ± 2°C with 40–60% humidity. All animal experiment protocols in this study were reviewed and approved by the Animal Care and Use Committee of Nantong University (Reference: S20210305-025) and were performed following the guidelines of the International Association for the Study of Pain.

### Drugs and administration

Agmatine sulfate salt (Cat#a7127), histamine (Cat#y0001779), compound 48/80 (Cat#C2313), resiniferatoxin (RTX, Cat#R8756), chlorpheniramine maleate (Cat#C3025), chloroquine (CQ, Cat#C6628), naloxone hydrochloride (Cat#N3136), amiloride hydrochloride (Cat#1019701) were all obtained from Sigma-Aldrich (St. Louis, MO, United States). MC903 (Cat# 2700/10) was purchased from R&D systems (Minneapolis, MN, United States). Morphine hydrochloride was obtained from China Northeast Pharmaceutical Group Shenyang No. 1 Pharmaceutical Co., Ltd (Shenyang City, China). Naloxone hydrochloride HC-030031 (Cat#2896), capsazepine (CPZ, Cat#0464) and APETx2 (Cat#4804) were purchased from Tocris (Minneapolis, MN, United States). ZnCl_2_ was obtained from National Pharmaceutical Group pharmaceutical Co., Ltd (Beijing, China). RTX and HC-030031 were freshly dissolved in 10% DMSO. Other reagents were dissolved in saline. Information relating to timing and doses are indicated in the results section or figure legends.

### Behavioral analysis

#### Establishing a neck model of acute itch in mice

Following a previously reported method (Green et al., 2006), mice were shaved at the nape of the neck and habituated to the testing environment for more than 2 days before experiments. On the day of behavioral testing, mice were individually placed in separate small plastic chambers (10 cm × 10 cm × 12.5 cm) on an elevated metal mesh for at least 30 min for habituation. Under brief anesthesia with isoflurane, mice were given an intradermal (i.d.) injection of 50 μL of drugs *via* a 26G needle into the nape of the neck. Following injection, mice were immediately returned to their chambers for video recording (30 min; Sony HDRCX610, Shanghai, China). The video was subsequently played back offline and scratching behavior was quantified in a blinded manner. One bout of scratching was recorded when a mouse lifted its hind-paw to scratch the application site and returned the paw back to the floor or mouth, regardless of the number of scratching strokes that were performed between these two movements.

#### Establishing a cheek model of acute itch in mice

A cheek model of acute itch was established as previously reported (Shimada and LaMotte, 2008), with minor modifications. The cheek of each mouse was shaved and the animal was habituated to the testing environment 2 days before experiments. On the day of behavioral testing, mice were moved to small plastic chambers (10 cm × 10 cm × 12.5 cm) on an elevated metal mesh and allowed at least 30 min for acclimation. Under brief anesthesia with isoflurane, mice were given an i.d. injection of 20 μL of drugs into the cheek. After the injection, the mice were immediately returned to the chambers and recorded for 30 min (Sony HDRCX610). The video was subsequently played back offline and both scratching behavior and wiping behavior were counted by an investigator who was blinded to the treatment groups. Scratching behavior was defined as lifting of the hind-paw to scratch the neck and the return of the paw back to the floor or mouth. Wiping behavior was defined as a lifting of the forelimbs toward the cheek for a time exceeding one or a few seconds before placing the forelimbs downward.

#### Tail flick test

The tail flick test was applied to determine heat pain sensitivity. In brief, the terminal 3 cm of the tail was immersed in a hot water bath at 52°C and the latency of rapid tail flick was recorded with a cutoff time of 10 s to avoid tissue injury.

#### Establishing a mouse model of MC903-induced atopic dermatitis

Before the start of the experiment, the neck of each mouse was depilated by depilatory cream. After 2 days of acclimatization, MC903 was diluted with absolute ethanol. Then, 3 nmol/30 μL of MC903 solution was applied to the neck skin of each mouse every day for 7 days. Control animals were treated with ethanol only. Spontaneous scratching behavior was quantified by recording 1 h of video at different time points indicated in figure legends. For the application of amiloride, 30 nM amiloride was injected intradermally into the affected neck skin of AD mice, spontaneous scratching behavior was quantified by recording 1 h of video before and after the application of amiloride.

#### Establishing a mouse model of imiquimod-induced psoriasis

Mice were modeled after 2 days of adaptation after depilation. The mice were given a daily topical dose of 62.5 mg of a commercially available imiquimod cream (Aldara™, 3 M Pharmaceuticals, United States) on the shaved neck skin every day for 7 days. Control mice were treated similarly with a control vehicle cream (Vaseline Lanette cream; Fagron, EU). Spontaneous scratching behavior was quantified by recording 1 h of video at different time points indicated in figure legends.

#### Western blotting analysis

Mice were terminally anesthetized with isoflurane 5 min after the injection of agmatine and the co-injection of agmatine and amiloride. Mice then were transcardially perfused with sterile saline. Dorsal root ganglia (DRG) were rapidly removed and homogenized in lysis buffer containing a cocktail of phosphatase inhibitors and protease inhibitors for total protein extraction assays. The protein concentrations were measured by the Pierce bicinchoninic acid (BCA) protein assay (Thermo Scientific, United States). Then, equal amounts of protein (40 μg) were loaded onto each lane, separated by 10% sodium dodecyl-sulfate polyacrylamide gel electrophoresis (SDS-PAGE) and transferred to PVDF membranes. After transfer, the membranes were blocked with 5% non-fat milk prepared with Tris–HCl Buffer Saline (TBS) at room temperature for 1 h and the PVDF membranes were incubated overnight at 4°C with primary monoclonal anti-p-ERK (mouse, 1:1000; Santa Cruz Biotechnology, United States) and primary monoclonal anti-ERK (mouse, 1:1000, Vazyme, China). The blots were washed and incubated with horseradish peroxidase-conjugated goat anti-mouse or goat anti-rabbit IgG secondary antibody (1:2000, Vazyme, China). Protein bands were visualized using an enhanced chemiluminescence detection kit (Thermo Scientific, United States) and the band densities were assessed and analyzed by NIH ImageJ software (NIH, Bethesda, MD, United States).

#### Cell culture

Dorsal root ganglion neurons were dissociated and prepared from mice (6–8 weeks-of-age) using a similar protocol as previously described (Wu et al., 2021). In brief, mice were decapitated after anesthesia with isoflurane. Lumbar DRGs were rapidly removed and placed in ice-cold oxygenated balanced D-Hank’s solution (Solarbio, Beijing, China). The DRGs were then digested in Dulbecco’s Modified Eagle Medium (DMEM, Gibco, Grand Island, NY, United States) containing collagenase D (0.6 U/ml; Roche, Mannheim, Germany) and dispase II (3.0 U/ml; Roche, Mannheim, Germany) for 35–40 min at 37°C. The ganglia were then triturated with fire-polished Pasteur pipettes. The dispersed cells were resuspended in F12 (Biological Industries, Beit HaEmek, Israel) medium supplemented with 10% FBS (Gibco, Waltham, MA, United States) and 1% penicillin/streptomycin (Biosharp, Hefei, China) and plated on coverslips coated with Poly-D-lysine (BBI Lifescience, Shanghai, China). Cell cultures were maintained in regular 95% air and 5% CO_2_ at 37°C in an incubator.

#### Patch clamping recordings

Dorsal root ganglion neurons were recorded 16–24 h after dissociation, as described previously (Wu et al., 2021). Small diameter DRG neurons (<25 μM) were chosen for whole-cell patch clamp recording in current-clamp mode or voltage-clamp mode at room temperature. Data were acquired using an EPC-10 amplifier (HEKA Eletronik, Germany) driven by a personal computer equipped with Pulse/PusleFit 8.3 software (HEKA Eletronik, Germany). DRG neurons were recorded with fire-polished, borosilicate glass patch pipettes (5–8 MΩ) which were pulled from borosilicate glass capillaries (Sutter Instrument, Novato, CA, United States) using a Sutter P-97 puller (Sutter Instrument, Novato, CA, United States). The bath solution consisted of 140 mM NaCl, 3 mM KCl, 2 mM MgCl_2_, 2 mM CaCl_2_, and 10 mM HEPES, pH 7.3. The pipette solution contained 30 mM KCl, 110 mM potassium gluconate, 0.5 mM EGTA, 5 mM HEPES, and 3 mM Mg-ATP, pH 7.3. Action potentials (APs) were evoked by depolarizing current steps with long- or short-time durations (1,000 ms or 2 ms). Long current steps were used to test AP rheobases and the number of evoked AP numbers was determined. Short current steps were used to trigger single action potentials to calculate the shape properties of APs. A gap-free protocol was used to record ongoing firing during the prefusion of agmatine. All signals obtained were sampled at 20 kHz, low-pass filtered at 5 kHz and analyzed offline.

#### Metabolite extraction and derivatization

The skin samples used for metabolite extraction and derivatization were collected from the nape of the neck from animals in the control, PSO and AD model groups, and stored at −20°C until use. An aliquot of each individual skin sample was weighed precisely and transferred to microcentrifuge tube. After the addition of 300 μL of extract solvent (precooled to −20°C and acetonitrile-methanol–water, 2:2:1), the samples were vortexed for 30 s, homogenized at 40 Hz for 4 min, and sonicated for 5 min in an ice-water bath. The homogenate and sonicate procedure was repeated three times, followed by incubation at −20°C for 1 h and centrifugation at 10,000 rpm and 4°C for 15 min. Derivatization was performed as follows: a 100-μL aliquot of the clear supernatant (or standard solution) was transferred to a microcentrifuge tube and then mixed with 50 μL of 20 mg/ml dansyl chloride in acetone and 50 μL of 100 mmol/l sodium carbonate after a 1 h incubation at 40°C in the dark. Dansyl derivatives were added to 50 μL of 1% formic acid in water. The samples were then vortexed for 30 s and centrifuged at 10,000 rpm and 4°C for 15 min. An 80-μl aliquot of the clear supernatant was transferred to an auto-sampler vial for ultra-high-performance liquid chromatography-tandem mass (UHPLC–MS/MS) analysis.

#### Agmatine measurement in the skin

The analysis of agmatine in samples of mouse skin was performed using UHPLC–MS/MS by Shanghai Biotree Biotech Co., Ltd. In brief, UHPLC separation was carried out using an Agilent 1,290 Infinity II series UHPLC System (Agilent Technologies) equipped with a Waters ACQUITY UPLC HSS T3 column (100 × 2.1 mm, 1.8 μM). Mobile phase A was 10 mmol/l ammonium acetate/formic acid and mobile phase B was acetonitrile. The column temperature was set to 35°C, the auto-sampler temperature was set to 4°C and the injection volume was 1 μL.

An Agilent 6,460 triple quadrupole mass spectrometer (Agilent Technologies), equipped with an AJS electrospray ionization (AJS-ESI) interface, was used for assay development. Typical ion source parameters were as follows: capillary voltage = +4,000/−3,500 V; nozzle voltage = +500/−500 V; gas (N_2_) temperature = 300°C; gas (N_2_) flow = 5 l/min; sheath gas (N_2_) temperature = 250°C; sheath gas flow = 11 l/min and nebulizer = 45 psi. The multiple- reaction monitoring (MRM) parameters for each of the targeted analytes were optimized using flow injection analysis by injecting standard solutions of the individual analytes, into the API source of the mass spectrometer. Several of the most sensitive transitions were used in the MRM scan mode to optimize the collision energy for each Q1/Q3 pair. Of the optimized MRM transitions per analyte, the Q1/Q3 pairs that showed the highest sensitivity and selectivity were selected as ‘quantifiers’ for quantitative monitoring. Additional transitions acted as ‘qualifiers’ for the purpose of verifying the identity of the target analytes. Agilent MassHunter Work Station Software (B.08.00, Agilent Technologies) was used for all MRM data acquisition and processing.

#### Statistical analysis

Data were analyzed using GraphPad Prism 6 (GraphPad Software Inc., United States), Origin 7.5 (OriginLab, United States) and Fitmaster v2X65 software (HEKA Electronik, Germany). All data are presented as mean ± standard error of the mean (S.E.M.). Statistical analysis was performed by the Student’s *t* test, one-way and two-way analysis of variance (ANOVA) followed by Dunnett multiple comparison tests. *p* < 0.05 was considered statistically significant.

## Results

### Agmatine induced acute itch in mice

**Figure 1:**
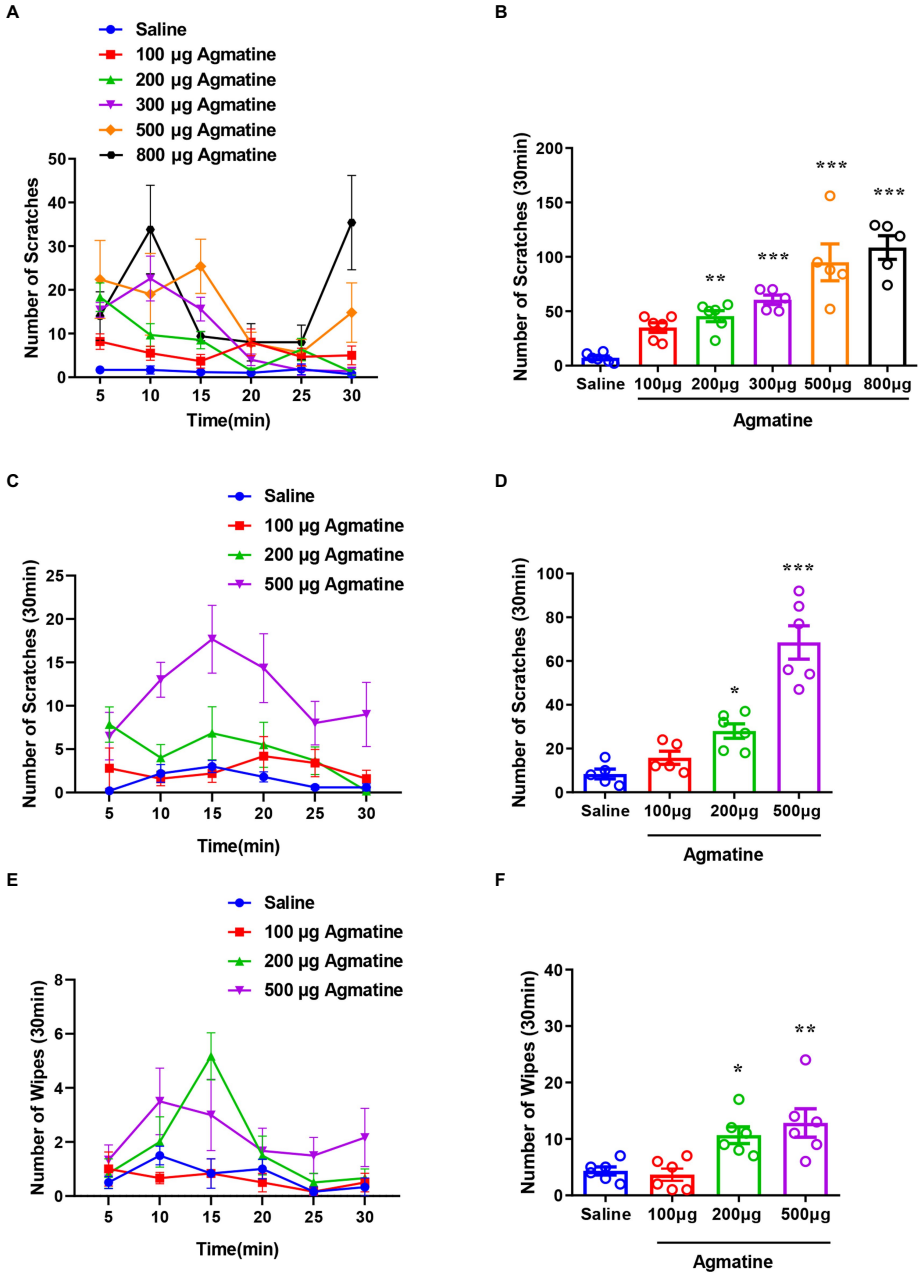
Agmatine evoked acute itch in mice. The time course **(A)** and the total number **(B)** of scratching behavior events within 30 min in response to an intradermal (i.d.) injection of agmatine (100–800 μg) in the nape of the neck in mice. The time course **(C)** and the total number **(D)** of scratching behavior events within 30 min of an i.d. injection of agmatine (100–500 μg) into the cheek of mice. The time course **(E)** and the total number **(F)** of wiping behavior events within 30 min of an i.d. injection of agmatine (100–500 μg) into the cheek of mice (*p < 0.05, **p < 0.01, ***p < 0.001 vs. Saline, one-way AVOVA following Bonferroni’s test; *n* = 5–6). All data are expressed by mean ± SEM.

To determine the *in vivo* effects of agmatine, we first investigated whether agmatine could directly induce itch behavior or not. An i.d. injection of agmatine (100–800 μg in 50 μL saline) into the nape of the neck was found to evoke scratching behavior in a dose-dependent manner [*F*_(5, 27)_ = 21.30, *p* < 0.0001; Figures 1A,B]. The cheek model showed that an i.d. injection of agmatine induced both itch-indicative scratching [*F*_(3, 19)_ = 31.31, *p* < 0.0001; Figures 1C,D] and pain-indicative wiping behaviors [*F*_(3, 20)_ = 8.165, *p* = 0.001; Figures 1E,F]. Notably, agmatine induced more itch-indicative scratching than pain-indicative wiping behavior (Figures 1D,F).

### The role of the μ-opioid receptor, histamine and capsaicin sensitive C-fibers in agmatine-induced itch

**Figure 2:**
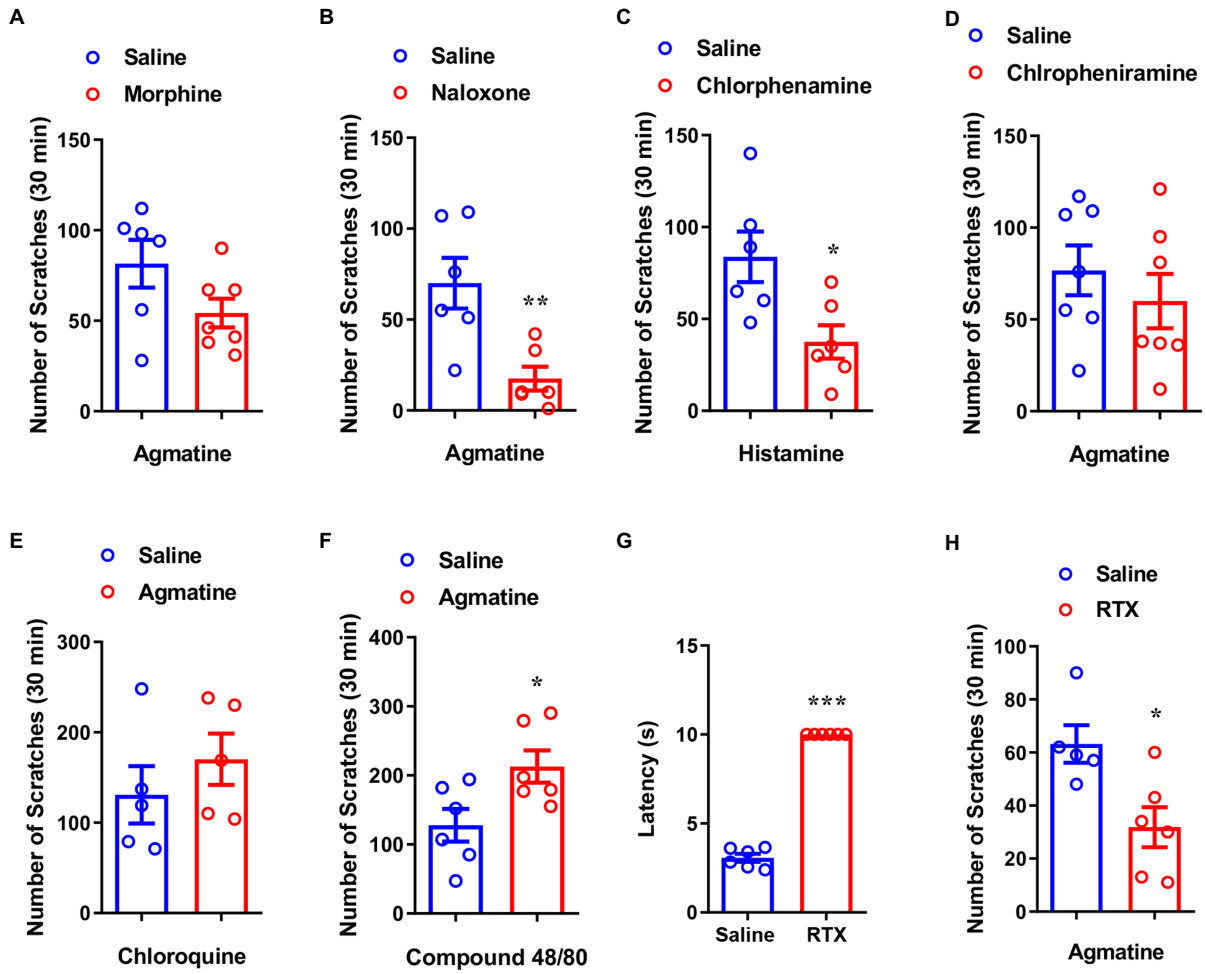
Capsaicin-sensitive nociceptors are required for agmatine-induced itch. The effects of morphine (1 mg/kg, i.p.) **(A)** and naloxone (1 mg/kg, i.p.) **(B)** on scratching behavior induced by agmatine (500 μg) in mice (*p < 0.05, **p < 0.01, ***p < 0.001 vs. agmatine, unpaired Student’s *t* test; *n* = 6–8 per group). **(C)** The effects of chlorpheniramine (1 mg/kg, i.p.) on scratching behavior induced by histamine in mice (*p < 0.05, **p < 0.01, ***p < 0.001 vs. histamine, unpaired Student’s *t* test; *n* = 6 per group). **(D)** The effects of the H1R antagonist chlorpheniramine (1 mg/kg, i.p.) on scratching behavior induced by agmatine (500 ug) in mice (*p < 0.05, **p < 0.01, ***p < 0.001 vs. agmatine, unpaired Student’s *t* test; *n* = 7). **(E)** Agmatine did not affect CQ-induced itch in mice (*p < 0.05, **p < 0.01, ***p < 0.001 vs. CQ, unpaired Student’s *t* test; *n* = 5 per group). **(F)** Agmatine significantly increased compound 48/80-induced itch in mice (*p < 0.05, **p < 0.01, ***p < 0.001 vs. compound 48/80, unpaired Student’s *t* test; *n* = 6 per group). **(G)** The tail flick time of RTX-treated mice in the tail flick test (*p < 0.05, **p < 0.01, ***p < 0.001 vs. Saline, unpaired Student’s *t* test; *n* = 6 per group). **(H)** The effects of RTX treatment on agmatine-induced itch in mice (*p < 0.05, **p < 0.01, ***p < 0.001 vs. agmatine, unpaired Student’s *t* test; *n* = 5–6 per group). All data are expressed by mean ± SEM.

The μ-opioid receptor is reported to be associated with morphine-induced itch (Nguyen et al., 2021; Wang et al., 2021). To further determine whether agmatine-induced behavior is modulated by μ-opioid receptor ligands, we applied morphine or naloxone by intraperitoneal injection (i.p.) injection 30 min before the administration of agmatine. We observed that morphine (1 mg/kg) did not affect agmatine-induced scratching behavior (t_11_ = 1.825, *p* = 0.0953; Figure 2A), but naloxone (1 mg/kg) significantly reduced agmatine-induced scratching behavior (t_10_ = 3.409, *p* = 0.0067; Figure 2B). These pharmacological intervention results suggested that agmatine induces itch in mice.

Because histamine is the principal itch mediator and is well known to cause experimental itch, we next used chlorphenamine, a blocker of the histamine H1 receptor (H1R), to identify whether agmatine-induced itch is histamine-dependent. Pretreatment with chlorphenamine significantly relieved histamine-induced scratching behavior (t_10_ = 2.807, *p* = 0.0186; Figure 2C) but had no significant effect on agmatine-induced itch (t_12_ = 0.8310, *p* = 0.4222; Figure 2D). The co-administration of agmatine did not affect CQ (100 μg)-induced histamine-independent itch (t_8_ = 0.9241, *p* = 0.3824; Figure 2E), but significantly aggravated compound 48/80 (50 μg)-induced histamine-dependent itch (t_10_ = 2.561, *p* = 0.0283; Figure 2F). Collectively, these results indicated that agmatine-induced itch is histamine-independent.

To understand how agmatine induced itch in mice, we next investigated whether capsaicin-sensitive nociceptors are required for agmatine-induced itch. Using resiniferatoxin (RTX) to chemically ablate capsaicin-sensitive C-fibers, we observed that RTX treatment significantly increased the withdrawal latency of tail-flick (t_10_ = 30.84, *p* < 0.0001; Figure 2G). Furthermore, agmatine-induced scratching behavior was also significantly ameliorated by RTX (t_9_ = 2.979, *p* = 0.0155; Figure 2H). These results suggested that capsaicin-sensitive C-fibers are required for agmatine-induced itch in mice.

### Acid-sensing ion channel 3 was required for agmatine-induced itch

**Figure 3:**
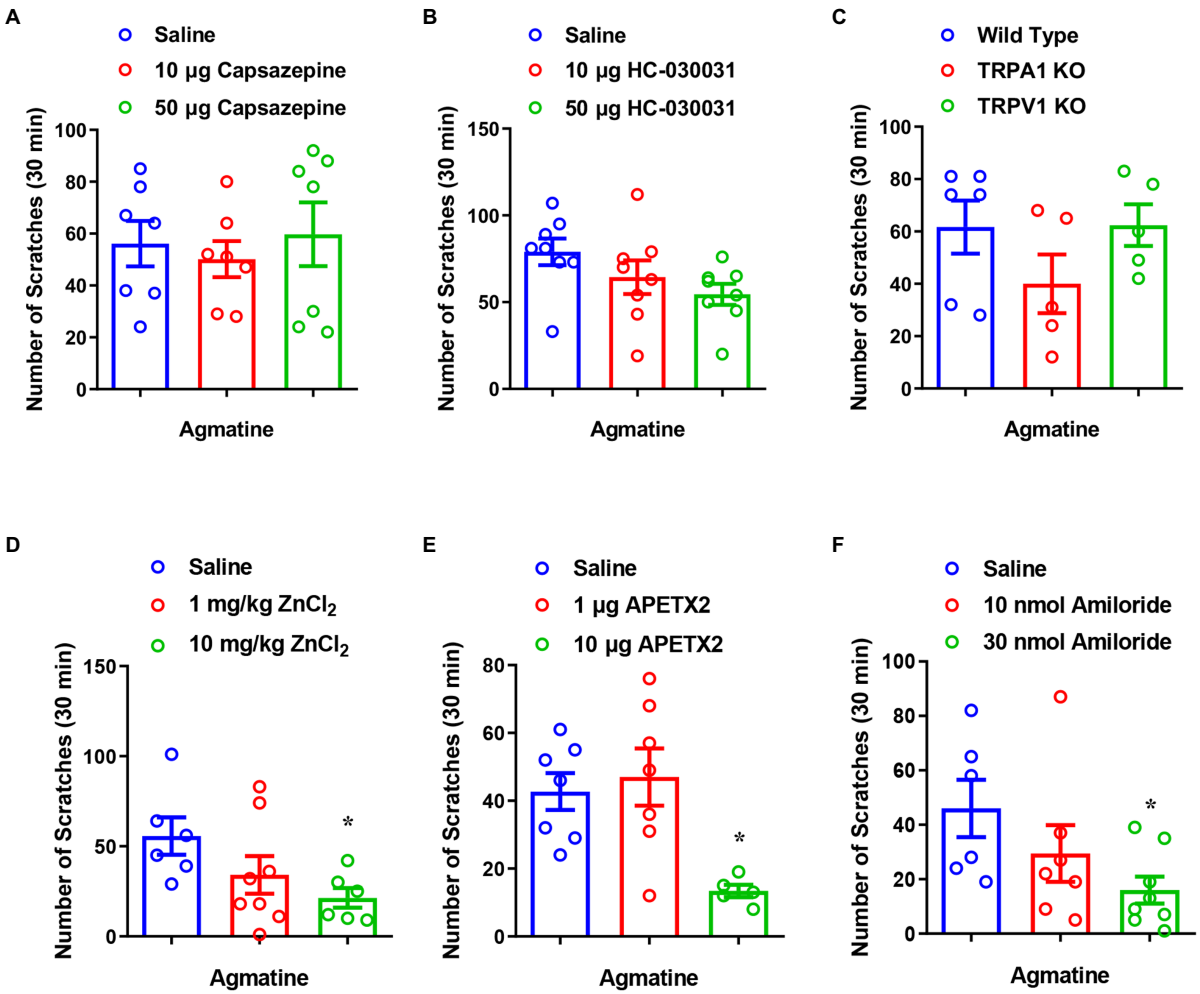
The inhibition of ASIC3, but not TRPA1 and TRPV1, reduced agmatine-induced itch. **(A,B)** The co-administration of agmatine (500 μg) and the TRPV1 blocker capsazepine (10–50 μg) or the TRPA1 blocker HC030031 (10–50 μg) on agmatine-induced itch in mice (*p* > 0.05 vs. agmatine, one-way AVOVA following Bonferroni’s test; *n* = 7–8 per group). **(C)** Agmatine-induced itch showed no obvious changes in TRPV1^−/−^ and TRPA1^−/−^ mice (*p* > 0.05 vs. Wild Type, unpaired Student’s *t* test; *n* = 5–6 per group). **(D–F)** The effects of the ASIC3 blocker ZnCl_2_ (1–10 mg/kg, i.p.) **(D)**, APETx2 (1–10 μg, i.d.) **(E)**, and amiloride (10–30 nmol, i.d.) **(F)** on agmatine (500 μg)-induced itch in mice (*p < 0.05 vs. Saline, unpaired Student’s *t* test; *n* = 6–7 per group). All data are expressed by means ± SEM.

Given that TRP channels (e.g., TRPA1 and TRPV1) are highly expressed in capsaicin-sensitive nociceptors and play a crucial role in itch signal transduction in DRGs (Clapham, 2003), we next investigated whether TRPA1 and TRPV1 channels participate in agmatine-induced itch. By pharmacological inhibition of these two channels, we found that the TRPV1 antagonist capsazepine [*F*_(2, 18)_ = 0.2541, *p* = 0.7784; Figure 3A] and the TRPA1 antagonist HC030031 [*F*_(2, 21)_ = 2.406, *p =* 0.1146; Figure 3B] had no significant effect on agmatine-evoked acute itch. Furthermore, agmatine-evoked itch behavior in TRPA1^−/−^ mice or TRPV1^−/−^ mice was similar to that observed in WT mice (t_9_ = 1.435, *p =* 0.1851 and t_9_ = 0.05518, *p* = 0.9572, respectively; Figure 3C). Collectively, these results suggest that capsaicin-sensitive C-fibers are required for agmatine-induced itch in mice, while TRPA1 and TRPV1 may be not involved in this process.

Acid-sensing ion channels are known to detect extracellular protons and contribute to peripheral sensory perception, including pain and itch sensations (Peng et al., 2015; Lee and Chen, 2018). We found that pre-administration of the ASIC3 blockers ZnCl_2_ (10 mg/kg; t_10_ = 2.933, *p =* 0.0150; Figure 3D), APETx2 (10 μg; t_10_ = 4.390, *p* = 0.0014; Figure 3E) and amiloride (30 nmol; t_12_ = 2.800, *p* = 0.0160; Figure 3F) significantly reduced agmatine-induced scratching behavior. These results indicate that ASIC3 activation is required for agmatine-induced itch.

**Figure 4:**
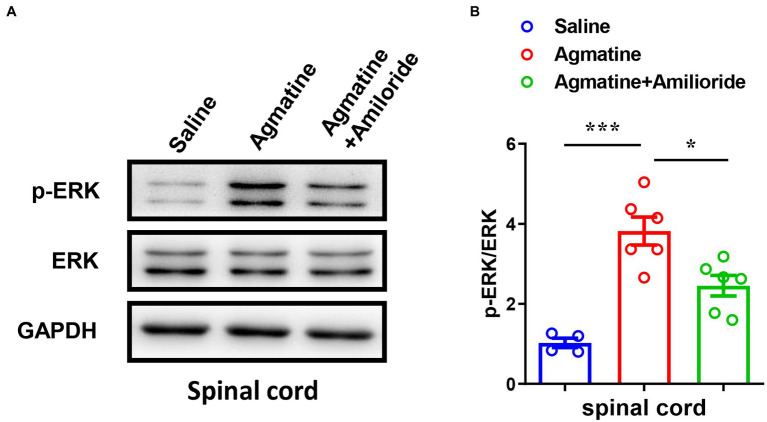
Activation of p-ERK in the spinal cord played a role in agmatine-induced itch in mice. **(A,B)** Western blotting **(A)** and quantification **(B)** revealed changes in the expression of p-ERK in the spinal cord 5 min after the i.d. injection of agmatine (*p < 0.05, ***p < 0.001 vs. Saline, unpaired Student’s *t* test; *n* = 4–6 per group) and an i.d. mixed injection of agmatine and amiloride (*p < 0.05 vs. agmatine, unpaired Student’s *t* test; *n* = 6 per group). All data are expressed by mean ± SEM.

Previous studies have shown that the phosphorylation of extracellular signal-regulated kinase (ERK) in DRGs and the spinal cord is involved in the signal transition of pain (Cheng and Ji, 2008) and itch (Zhang et al., 2014). Therefore, we next performed western blotting analysis and found that agmatine increased the expression levels of phosphorylation of ERK (p-ERK) in the spinal cord (t_8_ = 6.267, *p* = 0.0002; Figures 4A,B), whereas the ASIC3 blocker amiloride significantly reduced agmatine-induced p-ERK up-regulation in the spinal cord (t_10_ = 3.158, *p* = 0.0102; Figures 4A,B). The expression levels of p-ERK did not change significantly in DRG after the i.d. injection of agmatine (data not shown). Therefore, our results revealed that the activation of ASIC3 is required for the up-regulation of p-ERK induced by agmatine in the spinal cord of mice.

### Agmatine induced hyperexcitability in dorsal root ganglion neurons by activating acid-sensing ion channel 3

**Figure 5:**
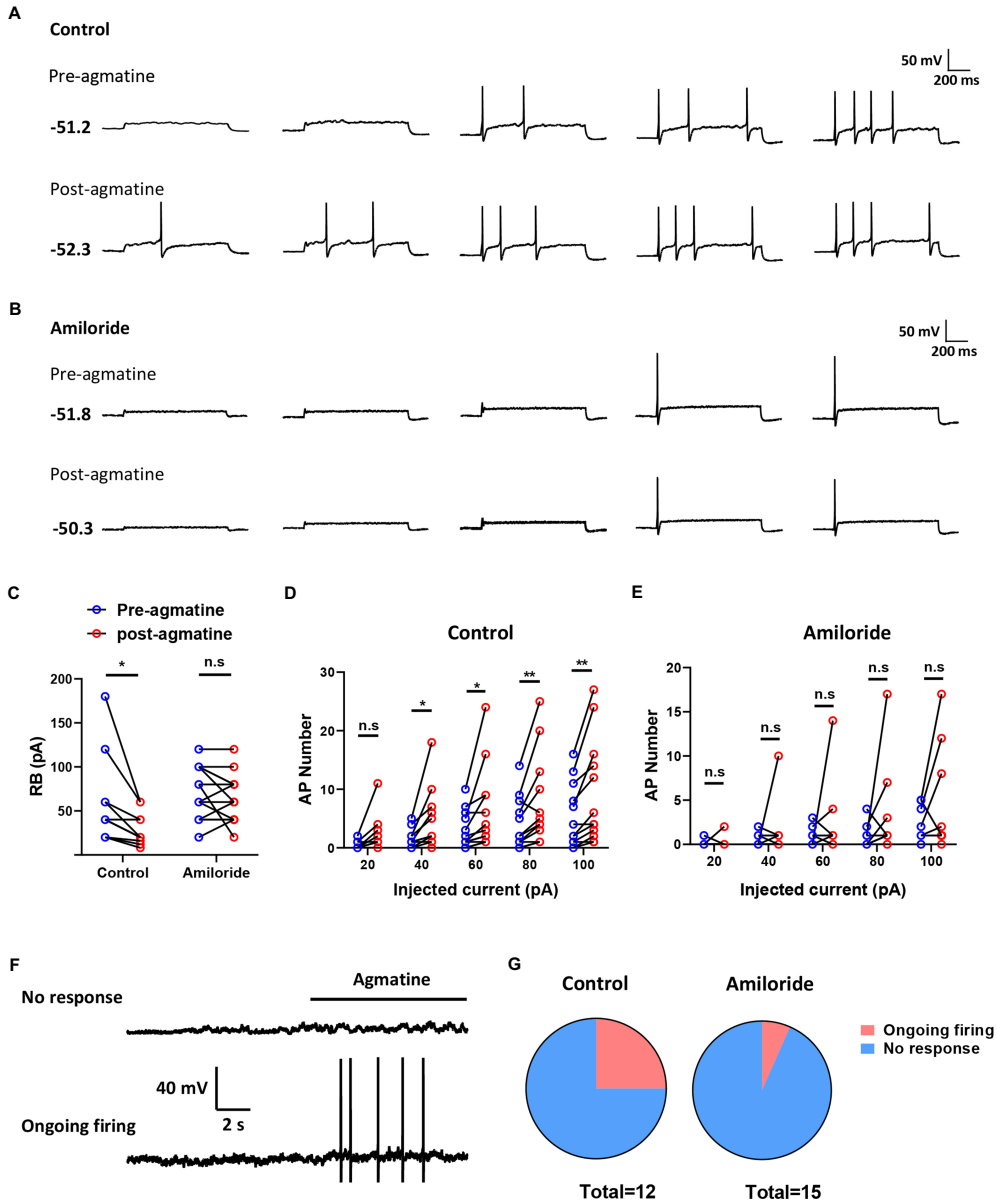
Agmatine induced spontaneous action potentials in some small-sized DRG neurons. **(A,B**) Examples of action potential (AP) traces evoked by current injection pre- and post-agmatine perfusion in small DRG neurons from control **(A)** and amiloride pre-incubation **(B)** groups. **(C)** Rheobase pre- and post-agmatine perfusion in small DRG neurons (*p < 0.05 post- vs. pre-, paired Student’s *t* test: *n* = 12 in control group, *n* = 15 in the amiloride pre-incubation group). **(D,E)** The number of APs pre- and post-agmatine perfusion in small DRG neurons (*p < 0.05, **p < 0.01 post- vs. pre-, two-way ANOVA following Bonferroni’s test: *n* = 12 in control group, *n* = 15 in the amiloride pre-incubation group). **(F)** Examples of traces during agmatine perfusion. **(G)** The proportion of DRG neurons exhibited ongoing firing during agmatine perfusion.

To determine whether agmatine has an effect on the excitability of DRG neurons and the role of ASIC3, we next performed patch-clamp recording in small DRG neurons (with a diameter less than 25 μM) *in vitro* from controls and an amiloride pre-incubation group (Figures 5A,B). Electrophysiological analysis demonstrated that membrane resistance and resting membrane potential did not change in response to 10 μM agmatine (Rm: t_11_ = 0.4026, *p* = 0.695; RMP: t_11_ = 0.2078, *p* = 0.8392; Table 1). However, current clamp recording showed that DRG neurons exhibited obvious hyperexcitability after agmatine (10 μM) perfusion, characterized as a significantly reduced rheobase after agmatine perfusion (t_11_ = 2.755, *p* = 0.0187; Figure 5C). Subsequently, agmatine significantly increased the number of action potentials trigged by depolarizing current steps (*F*_(4, 55)_ = 0.7217, *p* = 0.8284, *p* = 0.0418, *p* = 0.0185, *p* = 0.0098, and *p* = 0.0032 for 20 pA, 40 pA, 60 pA, 80 pA, and 100 pA current injections, respectively; Figure 5D). Consistent with the results derived from behavioral tests, pre-incubation with amiloride (100 μM) significantly abolished the effect of agmatine on DRG neurons. However, the rheobase and the number of trigged action potentials did not change significantly (Rheobase: t_14_ = 1.106, *p* = 0.2654; AP number: *F*_(4, 70)_ = 0.5278, *p* > 0.05 for each injected current; Figures 5C,E). In addition three neurons in the control group exhibited ongoing firing during the perfusion of agmatine, while only one of 15 cells still elicited ongoing firing following the administration of amiloride (100 μM; Figures 5F,G). Moreover, the properties of single APs such as amplitude, duration, afterhyperpolarization, the maximal rising slope and decaying slope did not change significantly in either group following treatment with agmatine (Table 1). Collectively, these data indicated that the activation of ASIC3 mediates agmatine-induced hyperexcitability in cultured DRG neurons from mice.

**Table 1:** Effects of *in vitro* agmatine prefusion on properties of membrane in mouse small DRG neurons from control group and amiloride preincubation group.

	Agmatine	Rm	RMP	RB	AMP	AHP	APD_50_	AHP_D50	S_rise	S_decy
Control (*n* = 12)	Pre-	1854.46 ± 187.53	−51.08 ± 1.63	56.67 ± 13.67	108.60 ± 3.09	−24.08 ± 1.16	2.56 ± 0.28	69.17 ± 7.98	207.35 ± 9.92	−61.44 ± 4.47
	Post-	1929.50 ± 181.50	−51.51 ± 2.03	29.67 ± 5.15*	109.78 ± 2.84	−22.77 ± 1.94	2.51 ± 0.22	60.52 ± 8.66	198.05 ± 13.30	−60.48 ± 5.27
Amiloride (*n* = 15)	Pre-	2630.39 ± 248.58	−51.48 ± 1.11	69.33 ± 7.78	110.20 ± 2.46	−23.22 ± 1.07	3.08 ± 0.28	88.69 ± 10.25	213.87 ± 13.08	−52.53 ± 3.27
	Post-	2652.25 ± 302.03	−54.60 ± 1.35	62.67 ± 7.00	110.64 ± 2.82	−22.25 ± 1.72	2.98 ± 0.20	78.07 ± 8.33	198.40 ± 11.24	−55.58 ± 3.57

Rm, membrane resistance; RMP, resting membrane potential; RB, rheobase; AMP, action potential amplitude; AHP, afterhyperpolarization amplitude; APD50, action potential duration at 50% of AMP; AHP_D50, afterhyperpolarization duration at 50% amplitude; S_rise, maximal rising slope; S_decay, maxmimal decaying slope; SEM, standard error of mean; *p < 0.05, paired Student’s *t*-test.

### The expression of agmatine was upregulated in a mouse model of MC903-induced atopic dermatitis

**Figure 6:**
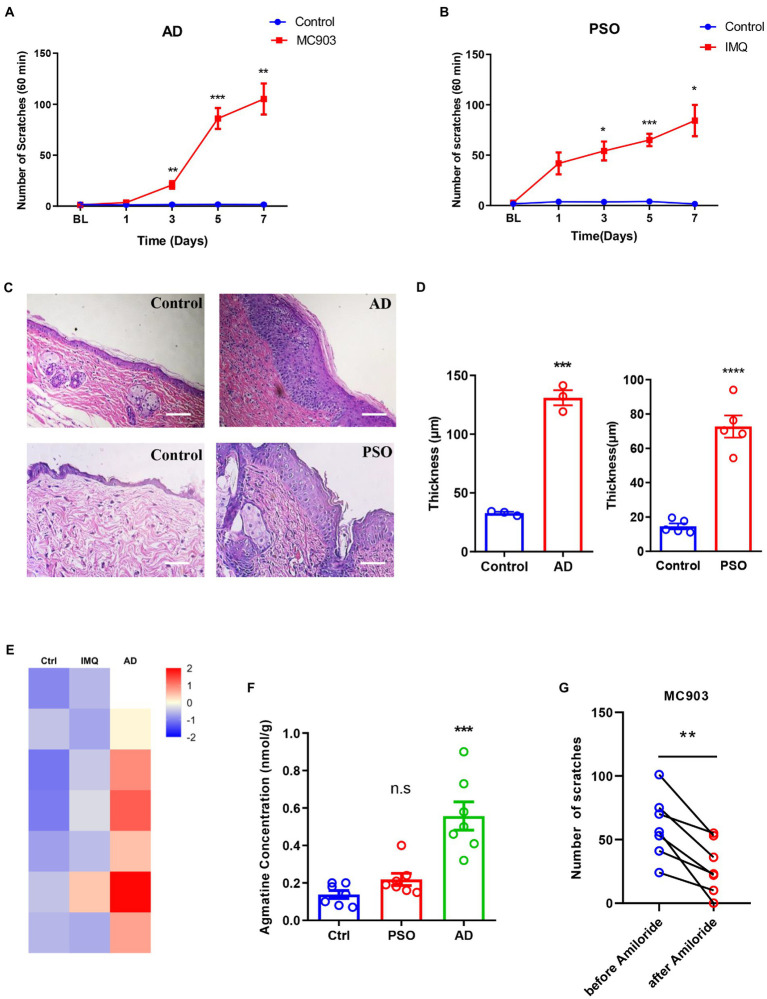
Up-regulation of agmatine in atopic dermatitis (AD)-induced itch. **(A,B)** The number of spontaneous scratching events in an hour on days 1, 3, 5, and 7 of the AD and PAO model mice. **(C)** Representative images of HE staining of the epidermis of AD and PSO model mice on day 7. Scale bars =50 μM. **(D)** Statistical analysis of epidermis thickness in AD and PSO model mice. Metabonomics heat map **(E)** and statistical analysis **(F)** of the concentration changes of agmatine in skin samples from AD and PSO models. **(G)** Blocking ASIC3 significantly reduced the scratching behavior of MC903-induced atopic dermatitis mice (*p < 0.05, **p < 0.01, ***p < 0.001, ****p < 0.0001 vs. Ctrl, two-way ANOVA, unpaired or paired Student’s *t* test; *n* = 3, 5, or 7 per group. The different colors in the metabonomics heat map represent the different concentration of agmatine in different skin samples, these are normalized values range from −2 to 2, for the value within −2 to 0, the deeper the colors is, the lower the concentration is; for the value within 0 to 2, the deeper the colors is, the higher the concentration is). All data are expressed by mean ± SEM. Ctrl, control; IMQ, imiquimod.

Finally, to investigate whether the expression levels of agmatine changed in chronic itch, we first established a mouse model of MC903-induced AD and a mouse model of imiquimod-induced psoriasis (PSO). Compared to the control group, the number of scratches within 60 min increased significantly from day3 to day7 in both itch models (Figures 6A,B). In addition, the epidermis thickness of both models was also significantly increased (t_4_ = 15, *p* = 0.0001 and t_8_ = 8.724, *p* < 0.0001, Figures 6C,D), thus suggesting that the mouse models of AD and PSO had been established successfully. Next, metabonomics analysis was performed to detect the concentration of agmatine in the affected skin under atopic dermatitis or psoriasis conditions. Compared with the control group, the concentration of agmatine in the affected skin was significantly increased in MC903-induced AD mice (t_12_ = 5.346, *p* = 0.0002; Figures 6E,F). However, in the imiquimod-induced PSO mouse model, the levels of agmatine showed no significant change (t_12_ = 2.096, *p* = 0.0608; Figures 6E,F). To further investigate whether ASIC3 also play a role in MC903-induced atopic dermatitis, we established previous AD mouse model, and blocked ASIC3 by intradermal injection of ASIC3 blocker amiloride (30 nM) at the affected neck skin, the result showed that blocking ASIC3 significantly reduced the scratching behavior of AD mice (t6 = 4.978, *p* = 0.0025, Figure 6G). Therefore, these results suggested that the increased levels of agmatine in the affected skin may play an important role in AD-induced pruritus, and ASIC3 is also required for MC903-induced induced itch behavior.

## Discussion

Itch is a common symptom in healthy conditions as well as many dermatological and systemic diseases. Chronic itch is a devastating clinical condition for clinicians to manage for which there are limited medications (Yang et al., 2022). In the present study, we identified and characterized agmatine as a novel mediator of itch by employing pharmacological and genetic manipulations, behavioral testing and patch clamp recording. First, we used a mouse model and found that an intradermal injection of agmatine in either the nape of the neck or cheek directly induced histamine-independent scratching behavior in a dose-dependent manner. We also confirmed that inhibition of the μ opioid receptor abolished agmatine-induced scratching. In addition, the ablation of capsaicin-sensitive C-fibers significantly abolished agmatine-induced scratching behavior. Furthermore, we showed that the pharmacological blockade or genetic knockout of TRPA1 or TRPV1 did not affect agmatine-induced itch, while ASIC3 blockers inhibited the pruritic effect of agmatine. Patch clamping recording showed that the perfusion of agmatine directly enhanced the neuronal excitability of dissociated small diameter DRG neurons. Consistently, the ASIC3 blocker amiloride reversed the neuronal hyperexcitability induced by agmatine and reduced the agmatine-induced upregulation of p-ERK in the spinal cord. Finally, we found that the levels of agmatine were elevated in the affected skin of a MC903-induced AD mouse model, and blocking ASIC3 significantly reduced the scratching behavior of AD mice. Collectively, our data revealed that the agmatine-induced activation of ASIC3 in DRG neurons contributed to itch signal transduction and may represent a novel therapeutic strategy for the clinical management of chronic itch.

### Agmatine is a novel non-histaminergic mediator of itch

Elucidation of the mechanism responsible for peripheral itch is beneficial for developing new topical treatments for itch, identifying potential pruritogens and determining relevant receptors or downstream ion channels. In the current study, we provide several pieces of evidence to support the use of agmatine as a novel mediator of itch. The increased levels of agmatine in the affected skin of mice with atopic dermatitis suggested that agmatine may be involved in the pathogenesis of atopic dermatitis-induced chronic itch. Notably, the levels of agmatine were not upregulated in imiquimod-induced psoriatic mice, thus suggesting that agmatine is not associated with psoriasis-induced chronic itch. Both atopic dermatitis and psoriasis are prevalent chronic inflammatory skin diseases that are caused by dysfunction of the immune system. Despite some similarity in their symptoms, there are differences in the mechanisms underlying atopic dermatitis and psoriasis. Psoriasis is mainly driven by Th17 cells and IL-17 (Rendon and Schakel, 2019), whereas atopic dermatitis is linked to Th2-associated inflammation, IL-4 and IL-13 (Chen et al., 2022). However, the factors responsible for the involvement of agmatine in atopic dermatitis, but not psoriasis-associated itch, has yet to be elucidated. A series of studies previously confirmed the neuroprotective role of agmatine in neurodegenerative diseases, including Parkinson’s disease, Alzheimer’s disease and epilepsy; these studies highlighted agmatine as a promising agent for the treatment of neurodegenerative diseases (Xu et al., 2018). In the present study, we demonstrated that the administration of agmatine may cause itch.

We demonstrated that agmatine-induced itch was histamine-independent. Our data showed that the ablation of capsaicin-sensitive C-fibers by RTX significantly abolished agmatine-induced scratching behavior in mice, thus suggesting that agmatine-induced itch is mediated by capsaicin-sensitive and nociceptive C-fibers. According to unbiased single-cell RNA sequencing (scRNA-seq), researchers previously defined 11 major subtypes of sensory neurons in DRGs (Usoskin et al., 2015; Hockley et al., 2019). Of these, three NP subtypes are likely to be itch-sensitive pruriceptors: NP1, which expresses Mrgprd; NP2, which expresses Mrgpra3 and Mrgprc11; and NP3, which expresses the interleukin-31 receptor A, IL-31r, natriuretic polypeptide B, Nppb and somatostatin (Sst; Zeisel et al., 2018; Sharma et al., 2020). MrgprA3/MrgprC11 neurons are responsive to various pruritic chemicals, including those that are well-known Mrgprs activators (chloroquine, BAM 8–22, and SLIGRL) and other receptor agonists (histamine, ET-1, and serotonin; Han et al., 2013; Meixiong and Dong, 2017). A recent study showed that piezo1 is expressed in abundance in Nppb^+^ neurons and is responsible for mechanical itch in mice (Hill et al., 2022). Another study reported that the population of itch-generating sensory neurons responsive to SL-NH2 (AKA PAR-2 activation peptide, a short mimetic agonist) *via* ASIC3 are Mrgpr11-positive neurons (Jiang et al., 2017).

### Acid-sensing ion channel 3 is indispensable for agmatine-induced itch

It was previously considered that TRPV1 mediated histamine-dependent itch while TRPA1 mediated histamine-independent itch. However, a recent study reported that TRPC3, but not TRPA1 or TRPV1, was associated with mitochondrial reactive oxygen species (mROS)-induced scratching behavior in mice, thus providing a potential therapeutic target for chronic itch (Kim et al., 2022). Because many painful inflammatory and ischemic conditions can lead to tissue acidosis, the study of acid signaling has predominantly focused on pain research since the discovery of acid-sensing of nociceptors (Lin et al., 2018). The expression levels of TDAG8, one of the acid sensing G-protein-coupled receptors (GPCRs), are known to increase after CFA-induced inflammation. The activation of TDAG8 can lead to the sensitization of TRPV1, thus highlighting the fact that proton-sensing GPCRs may be involved in acidosis-associated pain (Chen et al., 2009). Conversely, tissue acidosis caused by inflammation, infection, ischemia, hematomas or exercise is well recognized to activate nociceptors and produce pain which can be attenuated by amiloride, an ASIC blocker (Deval et al., 2008). Furthermore, inflammatory mediators, such as nerve growth factor (NGF), serotonin, interleukin-1, bradykinin, and brain-derived neurotrophic factor (BDNF) can increase the transcription of ASIC3, which perhaps contributes to the pain-enhancing effects of these mediators (Li and Xu, 2011). Several recent studies revealed that these proton-sensing ion channels also play crucial roles in itch. One study reported that TDAG8 is most abundant in Nppb^+^ DRG pruriceptors and potentiates the TRPV1-mediated calcium response *via* the Gs/cAMP/PKA pathway (Lin et al., 2017). Inflammation or dry skin treatment strongly enhances ASIC3 transcription, thus exacerbating the scratching behavior associated with chronic itch (Peng et al., 2015). Furthermore, the co-administration of chloroquine with the ASIC3 antagonist, APETx2, completely abolished chloroquine-evoked scratching in mice [35]. Collectively, these studies highlighted the role of ASIC3 in itch. Agmatine is similar to GMQ in structure and acts on the same non-proton-binding motif in the extracellular domain of ASIC3; subsequently, this can cause persistent activation of ASIC3 at a neutral pH or act synergistically with mild acidosis or reduced extracellular Ca^2+^ to activate ASIC3 and facilitate window currents (Li et al., 2010; Yu et al., 2010). Consistently, in the present study, we found that all ASIC3 blockers (ZnCl_2_, APETx2 or amiloride) could almost totally abolish agmatine-induced scratching behavior in mice. Although ASIC3 is considered to contribute to dry skin-induced itch (Peng et al., 2015), this is the first report of ASIC3 activation playing a role in atopic dermatitis associated itch. Thus, ASIC3 appears to be indispensable for agmatine-evoked itch in mice.

ERK activation in the spinal dorsal horn occurs in response to stimuli that are strong enough to cause C-fibers to fire (Ji et al., 1999). Previous studies showed that phosphorylation of ERK in the spinal cord is required for histamine-dependent itch in mice (Zhang et al., 2014; Huang et al., 2018). Although we found that agmatine induces histamine-independent itch, we also detected upregulated expression of p-ERK in the spinal cord 5 min after an intradermal injection of agmatine in mice. These results suggest that agmatine may activate capsaicin-sensitive C-fibers and transmit itch signals to the spinal cord. Furthermore, patch clamp recording analysis showed that 3 of 12 cells exhibited ongoing firing during the perfusion of acute dissociated DRG neurons with agmatine. Our results also indicate that the phosphorylation of ERK in the spinal cord may contribute not only to histamine-dependent itch but also to histamine-independent itch. A previous study showed that increased levels of p-ERK in the dorsal spinal cord were sustained in chronic itch models induced by squaric acid dibutylester, imiquimod and 2, 4-dinitrofluorobenzene (DNFB; Huang et al., 2018; Liu et al., 2021). The phosphorylation of ERK may subsequently lead to increased excitability of the spinal cord neurons; this may be a possible mechanism responsible for the conversion from acute itch to chronic itch.

### Agmatine induced hyperexcitability of dorsal root ganglion neurons *via* the activation of acid-sensing ion channel 3

Unlike the common pruriceptors, such as histamine receptors and a number of Mrgpr family members (for example MrgprA3, MrgprA1 and MrgprC11), ASIC3 contributes to both itch and pain. This led us to hypothesize whether there was a relationship between acidosis-associated itch and nociception. By applying the cheek itch model, we verified that agmatine caused itch (scratching) as well as pain (wiping). However, pharmacological inhibition of the μ receptor alleviated agmatine-induced scratching whereas μ receptor activation had no effect, thus suggesting that agmatine acts as a pruritogen, but not an algogen. Our results are consistent with a recent study that reported that the direct activation of ASIC3 by SL-NH2 causes itch rather than pain by evoking MrgprC11-expressing neurons (Peng et al., 2015). Although agmatine reduced the rheobase of action potential firing in 10 of the 12 neurons, our results also showed that agmatine induced ongoing firing in approximately 25% of DRG neurons. When pre-incubated with amiloride, this percentage dropped to only 6.7%, thus suggesting that ASIC3 is involved in agmatine-evoked ongoing firing. Moreover, it is possible that there are specific cell types that mediate ASIC3-dependent itch relative to pain. Nevertheless, whether the subpopulation of neurons that are responsive to SL-NH2 *via* ASIC3 is the same to as that responsible for agmatine-induced itch has yet to be elucidated. Future studies need to identify the population of DRG neurons that evokes firing by agmatine.

In a word, we have provided strong evidence to indicate that agmatine is a novel mediator of itch and that ASIC3 is involved in agmatine-induced histamine-independent pruritus in mice. In addition, our findings reveal the importance of treating chronic pruritus by targeting neuronal ASIC3 signaling.

## Data availability statement

The original contributions presented in the study are included in the article/supplementary material, further inquiries can be directed to the corresponding authors.

## Author contributions

G-KZ, W-JX, YL, YZ, C-ZF, J-TZ, S-YS, R-MW, TL, and BW contributed to the work design, performed experiments, and analyzed and interpreted data from all the experiments. G-KZ, W-JX, and YL were performed the animal behavior experiments. BW performed the electrophysiological recording. YZ, C-ZF, J-TZ, and G-KZ were performed the molecular biology experiments. G-KZ, BW, and TL wrote and completed the manuscript. All authors critically revised and approved the final manuscript and agreed to take the responsibility for all aspects of the study.

## Funding

This work was supported by the National Natural Science Foundation of China (82171229 and 81870874 to TL and 82101305 to BW), Natural Science Research Project of Colleges and Universities in Jiangsu Province of China (20KJB310019 to BW), Natural Science Foundation of Jiangsu Province of China (BK20210839 to G-KZ), and Basic Science Research Program of Nantong City, Jiangsu Province of China (JC2021007 to G-KZ).

## Conflict of interest

The authors declare that the research was conducted in the absence of any commercial or financial relationships that could be construed as a potential conflict of interest.

## Publisher’s note

All claims expressed in this article are solely those of the authors and do not necessarily represent those of their affiliated organizations, or those of the publisher, the editors and the reviewers. Any product that may be evaluated in this article, or claim that may be made by its manufacturer, is not guaranteed or endorsed by the publisher.
